# Bridging Age-Friendly Health Systems Into the Community

**DOI:** 10.1093/geroni/igab046.1026

**Published:** 2021-12-17

**Authors:** Erin Emery-Tiburcio, Rani Snyder

## Abstract

As the Age-Friendly Health Systems initiative is implemented across the country, building bridges into the community assures that older adults live safely, enjoy good health and stay involved in their communities. In this symposium, we present innovative approaches that bridge the Age-Friendly Health Systems initiative into the community. Each presentation explores how the 4Ms concepts are integrated into their programs and enhance community with older adults. CATCH-ON Connect provides free cellular-enabled tablets and individual training to older adults to help them do What Matters to them by using their tablets. Rush@Home is a home-based primary care program that addresses the 4Ms in the comfort of an older adult’s home. Social Connections was co-designed with older adults to decrease loneliness by connecting older adults to each other. The Caregiver Initiative identifies and supports caregivers of older adults by meeting the 4Ms health needs of both caregiver and care recipient. Dementia Friendly Communities engage community stakeholders in a process to become educated, creating safe and respectful environments for individuals with dementia. By exploring these approaches, we can bridge the Age-Friendly Health Systems initiative into the community to support older adults outside the four walls of the health system.

